# Systematic analysis of the expression profile of non-coding RNAs involved in ischemia/reperfusion-induced acute kidney injury in mice using RNA sequencing

**DOI:** 10.18632/oncotarget.22130

**Published:** 2017-10-26

**Authors:** Jun Zhou, Hongtao Chen, Youling Fan

**Affiliations:** ^1^ Department of Anesthesiology, The First People’s Hospital of Foshan, Foshan, Guangdong Province 528000, China; ^2^ Department of Anesthesiology, Eighth People’s Hospital of Guangzhou, Guangzhou, Guangdong Province 510060, China; ^3^ Department of Anesthesiology, Panyu Central Hospital, Guangzhou, Guangdong Province 511400, China

**Keywords:** ischemia/reperfusion, acute kidney injury, non-coding RNAs, sequencing data analysis, expression profiles difference

## Abstract

Acute kidney injury (AKI) is a common and serious disease characterized by a rapid decline in renal function and has an unacceptably high mortality rate with no effective treatment beyond supportive care. AKI can be induced by many factors such as ischemia/reperfusion (IR), sepsis, and drug-induced nephrotoxicity. However, the molecular mechanisms of AKI are poorly understood. A non-coding RNA (ncRNA) is a RNA molecule that is not translated into a protein. NcRNAs play multiple roles in cellular processes, and mutations or imbalances of these molecules within the body can cause a variety of diseases. Although growing evidence has supported the key role of ncRNAs in AKI, the specific mechanism remains largely unknown. In this study, the second-generation gene sequencing was performed to investigate the expression patterns of ncRNAs, including microRNA (miRNA), long non-coding RNAs, and circular RNAs, in the kidneys of mice subjected to IR-induced AKI. This information will contribute to future research of the mechanism of ncRNAs in the pathogenesis of AKI and facilitate the identification of novel therapeutic targets of ncRNAs.

## INTRODUCTION

Acute kidney injury (AKI) is a major clinical problem without an effective therapy [1, 2]. Renal ischemia/reperfusion (IR) injury, along with sepsis and nephrotoxin injury, is the leading cause of AKI in perioperative patients [3, 4]. The prognosis of AKI is poor because there are no currently available therapies to effectively treat or prevent IR-induced AKI [5, 6]. However, the mechanism underlying IR-induced AKI has not been fully elucidated. Therefore, it is urgent to explore its pathogenesis to develop an effective treatment for IR-induced AKI.

Non-coding RNAs (ncRNAs) are a family of RNA molecules that typically do not code proteins but regulate gene expression, thus involving themselves in diverse cellular processes such as development, cell differentiation and proliferation, cell cycle, apoptosis, and metabolic function [7–10]. Based on their size, ncRNAs are subdivided into small ncRNAs (<200 nucleotides long), which encompass microRNAs (miRNAs), long ncRNAs (lncRNAs) with a length between 0.2 and 2 Kb, and circular RNAs (circRNA), which consist of a closed continuous loop [11]. Moreover, emerging data have demonstrated that ncRNAs are critically involved in the pathogenesis of AKI, particularly in IR-induced AKI [12–14]. However, the regulatory functions of ncRNAs in AKI and their underlying functional mechanisms have not been systematically described. Therefore, comprehensive estimations and analyses of the ncRNAs underlying the pathogenesis of AKI are essential to develop effective strategies to treat this troublesome disorder and prevent its progression.

In this study, we utilized an RNA sequencing approach to investigate ncRNAs in the kidneys of mouse subjected to IR-induced AKI. Our study is designed to systematically identify the expression profiles of non-coding RNAs involved in IR-induced AKI and to provide a valuable resource for exploring their functional roles in AKI therapy, that the raw data in this study can be available in NCBI SRA database.

## RESULTS

### IR-induced AKI

**Figure 1:**
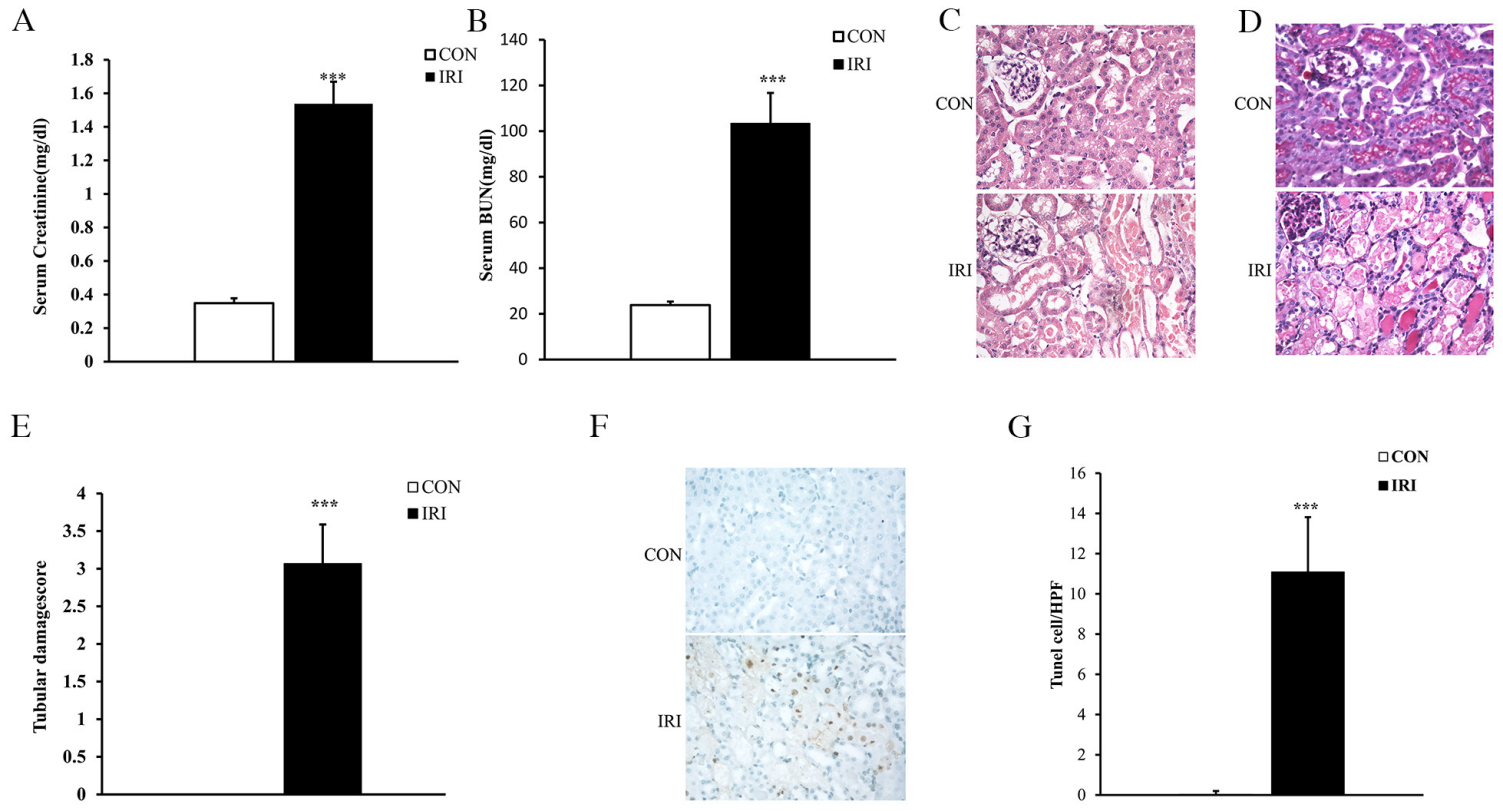
Ischemia/reperfusion induces AKI **(A)** Effect of either ischemia or control treatment on serum creatinine in mice at 24 hours after surgery. ^***^p<0.001 vs. CON group, n=6 per group. **(B)** Effect of either ischemia or control treatment on serum BUN in mice at 24 hours after surgery. ^***^p<0.001 vs. CON group, n=6 per group. **(C)** Representative photomicrographs of HE-stained kidney sections from mice at 24 hours after either IRI or control treatment. (Original magnification: ×400). **(D)** Representative photomicrographs of PAS-stained kidney sections from mice at 24 hours after either IRI or control treatment. (Original magnification: ×400). **(E)** Quantitative assessment of tubular damage based on PAS staining of sections from mice at 24 hours after IRI treatment. ^***^p<0.001 vs. CON group, n=6 per group. **(F)** Representative photomicrographs of kidney sections from mice at 24 hours after either ischemia or control treatment. The sections were stained for apoptotic cells (brown) and counterstained with methyl green (green). (Original magnification: ×400). **(G)** Quantitative analysis of apoptotic cells in the kidneys from mice at 24 hours after ischemia or control treatment. ^***^p<0.001 vs. CON group, n=6 per group. HPF, high-power field; TUNEL, terminal transferase dUTP nick-end labeling.

There is much evidence indicating that IR is the leading cause of AKI [15, 16]. To determine the effect of IR on AKI, kidney function was evaluated at 24 hours after IR treatment. Renal function was relatively deteriorated in mice in the IRI group, with blood creatinine and urea nitrogen levels that were markedly higher than those in mice in the CON group (Figure 1A and 1B). Consistent with the deterioration of kidney function in mice subjected to IR treatment, there was substantial exacerbation in the histological injury of the kidneys as shown by more tubular epithelial cell injury, tubular dilation, and intratubular cast formation in mice in the IRI group compared with mice in the CON group (Figure 1C, 1D and 1E).

### Cell apoptotic in IR-induced AKI

Increasing evidence has indicated that tubular necrosis/apoptosis is an important mechanism underlying IR-induced AKI [17, 18]. Therefore, we investigated tubular epithelial cell apoptosis induced by IR to confirm the success of the model. Our results showed that the number of apoptotic tubular cells significantly increased in kidneys from mice subjected to IR treatment as assessed by TUNEL staining (Figure 1F and 1G).

### Differentially expressed (DE) ncRNAs and mRNAs

**Figure 2:**
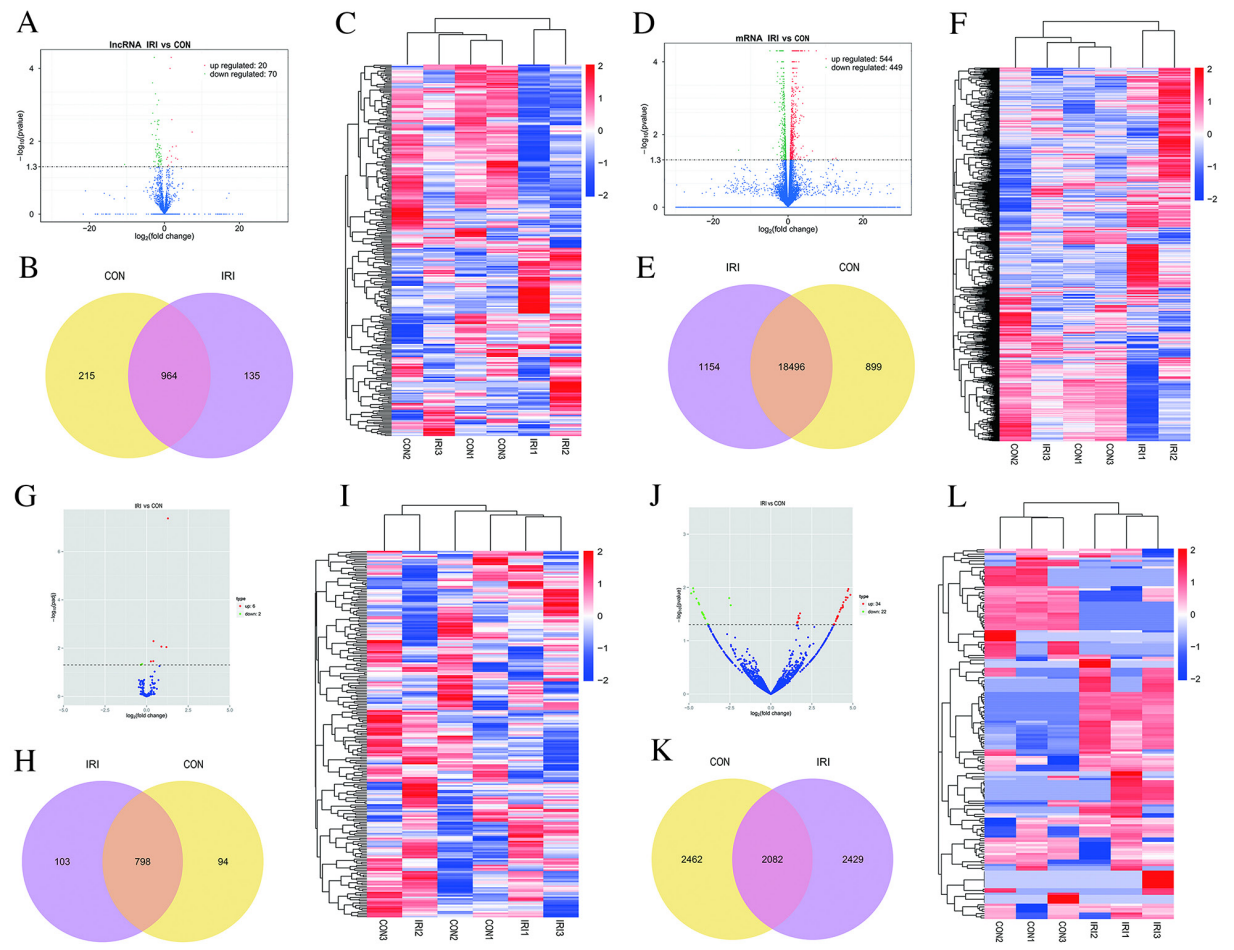
Changes in the expression profile of lncRNAs, mRNAs, miRNAs and circRNAs in the kidneys of mice subjected to IR Volcano plots indicate the respective up-regulated and down-regulated lncRNAs, mRNAs, miRNAs and circRNAs in mice from the IRI group compared with the CON group **(A, D, G** and **J)**. Venn diagrams showing the respective number of overlapping lncRNAs, mRNAs, miRNAs and circRNAs in the IRI group compared with the CON group **(B, E, H** and **K)**; Heat maps showing the respective hierarchical clustering of changed lncRNAs, mRNAs, miRNAs and circRNAs in mice from the IRI group compared with those from the CON group **(C, F, I** and **L)**. In the clustering analysis, up-regulated and down-regulated genes are colored in red and blue, respectively.

To determine if ncRNAs are involved in the pathogenesis of IR-induced AKI, we analyzed DE ncRNAs and mRNAs using significance analysis of sequencing technique based on a q-value <0.05. DE ncRNAs and mRNAs in the kidney samples between mice in the IRI group and CON group are shown as a volcano plot, Venn diagram and clustering map. Information regarding the top 20 up-regulated and 20 down-regulated lncRNAs, mRNAs, miRNAs and circRNAs in the kidney tissues of mice in the IRI group compared with the CON group are listed in Tables 1-4, respectively. The full list is presented in the table if number of DE ncRNAs was less than 20. The complete sequence data can be obtain in the NCBI database (Accession: SRP107607). Figure 2A-2C show the volcano plot, Venn diagram and clustering map of DE lncRNAs, respectively, and Figure 2D-2F show the volcano plot, Venn diagram and clustering map of DE mRNAs, respectively. Figure 2G-2I show the volcano plot, Venn diagram and clustering map of DE miRNAs, respectively. Figure 2J-2L show the volcano plot, Venn diagram and clustering map of DE circRNAs, respectively. The results of the DE ncRNAs were as follows. There were 90 DE lncRNAs (20 up-regulated and 70 down-regulated), 8 DE miRNAs (6 up-regulated and 2 down-regulated) and 56 DE circRNAs (34 up-regulated and 22 down-regulated) in the kidneys of mice in the IRI group compared with those in the CON group. The results of the DE mRNAs indicated 993 DE mRNAs (544 up-regulated and 449 down-regulated) in the kidneys of mice in the IRI group compared with those in the CON group.

**Table 1:** The detail information of the top 20 up-regulated and 20 down-regulated lncRNAs

Gene_id	Gene_name	Gene location	IRI_FPKM	CON_FPKM	Log2 (foldchange)	P value	Regulation
XLOC_024803	-	chr2:167846635-167847896	36.4741	10.4918	1.79761	0.00005	up
ENSMUSG035570R186775.6	Snhg7os	chr2:26643314-26645944	1.02682	0	/	0.00005	up
ENSMUSG035570R186320.1	Gm12840	chr4:117700187-117700923	54.1934	19.0378	1.50925	0.0001	up
XLOC_004488	-	chr10:27898276-27987459	0.977554	0	/	0.0025	up
XLOC_016913	-	chr16:86287077-86288750	2.67069	0.672917	1.98871	0.00255	up
XLOC_045083	-	chrX:19700403-19707577	4.4814	0.0268362	7.38363	0.0056	up
XLOC_028252	-	chr4:35474640-35550758	2.54105	0	/	0.00645	up
XLOC_007723	-	chr11:96133785-96165451	17.7686	2.03496	3.12626	0.0136	up
XLOC_028578	-	chr4:77310995-77315061	0.834581	0.187563	2.15368	0.01425	up
XLOC_034449	-	chr6:50535592-50538023	1.87554	0.716619	1.38803	0.01845	up
ENSMUSG035570R199848.1	Gm29337	chr1:88868474-88875410	1.7262	0	/	0.0212	up
XLOC_014293	-	chr15:61157813-61166426	0.589792	0.160592	1.87681	0.02555	up
XLOC_025413	-	chr3:56956492-57033019	1.05425	0.588352	0.84147	0.02765	up
ENSMUSG035570R185562.6	2610028E06Rik	chr4:125890414-125917171	0.869271	0.108219	3.00586	0.0292	up
XLOC_011927	-	chr13:112441962-112447266	0.580414	0.0513891	3.49755	0.03115	up
ENSMUSG035570R192274.2	Neat1	chr19:5843680-5845259	107.377	70.0475	0.616277	0.0312	up
ENSMUSG00000103476.1	Gm34302	chr3:63481111-63483879	3.88583	0	/	0.0355	up
XLOC_027979	-	chr3:145629486-145632977	1.3193	0.37305	1.82233	0.0373	up
XLOC_010884	-	chr13:112100741-112102257	2.22888	1.04228	1.09658	0.0456	up
XLOC_014251	-	chr15:52183833-52187198	2.34821	1.29478	0.858857	0.04985	up
ENSMUSG 035570R174469.6	Gm15348	chr8:12706943-12719127	0.576011	3.74057	-2.69909	0.00005	down
ENSMUSG 00000100426.1	Gm4208	chr1:62821354-62832370	3.76159	24.1171	-2.68064	0.00005	down
ENSMUSG 035570R184923.1	Gm15611	chr5:8998401-8999669	3.00868	26.4068	-3.13371	0.00025	down
ENSMUSG 035570R186474.2	9130204K15Rik	chr11:79781978-79782887	0.940965	4.95249	-2.39594	0.0005	down
ENSMUSG 035570R198747.6	Gm27216	chr9:83240480-83254540	9.35815	27.3057	-1.54491	0.00075	down
XLOC_019356	-	chr18:62028223-62032013	3.81839	14.6974	-1.94452	0.001	down
XLOC_040399	-	chr7:119724316-119760759	1.23921	10.5862	-3.09469	0.0017	down
XLOC_015796	-	chr16:20867025-20876666	0.420567	1.13211	-1.4286	0.00265	down
XLOC_023010	-	chr2:174019236-174022851	0.265096	1.56356	-2.56025	0.00285	down
XLOC_031196	-	chr5:17141930-17182151	0.585445	2.01737	-1.78487	0.0029	down
XLOC_014584	-	chr15:88851298-88853645	0.524277	1.82643	-1.80063	0.0034	down
ENSMUSG 035570R184854.1	Gm12678	chr4:44943752-44948330	4.8818	14.4658	-1.56716	0.0035	down
XLOC_040791	-	chr8:12749193-12752563	0.630367	2.26365	-1.84439	0.0047	down
XLOC_018704	-	chr17:45866182-45868006	0.0965331	0.892256	-3.20836	0.0066	down
XLOC_040818	-	chr8:13950530-13975032	2.12566	10.3192	-2.27935	0.00695	down
XLOC_013409	-	chr14:46239989-46243131	0.735642	2.01649	-1.45477	0.00885	down
ENSMUSG 00000101746.1	2310043L19Rik	chr1:177641541-177642943	0.499305	2.23931	-2.16506	0.00895	down
ENSMUSG 035570R192525.1	Gm20461	chr17:34640845-34643977	0	1.72738	/	0.01015	down
XLOC_030332	-	chr4:107433666-107434431	0.781205	2.86033	-1.87241	0.0118	down
ENSMUSG 035570R187343.1	1700021N21Rik	chr4:134448765-134450171	0.547932	1.78906	-1.70714	0.012	down

**Table 2:** The detail information of the top 20 up-regulated and 20 down-regulated mRNAs

Gene_id	Gene_name	Gene location	IRI_FPKM	CON_FPKM	Log2(foldchange)	P value	Regulation
ENSMUSG035570R129380	Cxcl1	chr5:90891240-90893115	29.698	2.12987	3.80153	0.00005	up
ENSMUSG035570R126822	Lcn2	chr2:32384632-32388252	19.5004	2.64261	2.88347	0.00005	up
ENSMUSG035570R105355	Casp14	chr10:78711996-78718293	0.507357	0	/	0.00005	up
ENSMUSG035570R174115	Saa1	chr7:46740500-46742980	2.95328	0	/	0.00005	up
ENSMUSG035570R140322	Slc25a24	chr3:109123148-109168457	10.4259	2.77237	1.91098	0.00005	up
ENSMUSG035570R101228	Uhrf1	chr17:56303320-56323486	7.8469	1.77623	2.14331	0.00005	up
ENSMUSG035570R128885	Smpdl3b	chr4:132732965-132757252	8.7952	1.63025	2.43162	0.00005	up
ENSMUSG035570R169516	Lyz2	chr10:117277333-117282274	88.7595	36.0341	1.30054	0.00005	up
ENSMUSG035570R114846	Tppp3	chr8:105467492-105471526	11.4114	1.18354	3.2693	0.00005	up
ENSMUSG035570R127875	Hmgcs2	chr3:98280434-98310738	89.6695	7.32741	3.61324	0.00005	up
ENSMUSG035570R124164	C3	chr17:57203969-57228136	30.6048	3.17806	3.26754	0.00005	up
ENSMUSG035570R153746	Ptrh1	chr2:32775785-32784428	17.0299	4.18731	2.02397	0.00005	up
ENSMUSG035570R113584	Aldh1a2	chr9:71215788-71296243	5.96977	1.29452	2.20526	0.00005	up
ENSMUSG035570R161947	Serpina10	chr12:103614785-103631444	12.3372	1.09818	3.48982	0.00005	up
ENSMUSG035570R151439	Cd14	chr18:36725073-36726736	19.0606	7.3606	1.3727	0.00005	up
ENSMUSG035570R140152	Thbs1	chr2:118111875-118127133	22.5987	10.4937	1.10671	0.00005	up
ENSMUSG035570R122146	Osmr	chr15:6813576-6874969	10.5415	4.1118	1.35824	0.00005	up
ENSMUSG035570R128494	Plin2	chr4:86656564-86670060	66.343	23.9992	1.46696	0.00005	up
ENSMUSG035570R178597	Cyp4a12b	chr4:115411623-115439034	8.19422	1.65673	2.30627	0.00005	up
ENSMUSG035570R105667	Mthfd2	chr6:83305690-83325908	6.77719	2.25819	1.58552	0.00005	up
ENSMUSG035570R133715	Akr1c14	chr13:4049010-4090422	85.8208	360.919	-2.07228	0.00005	down
ENSMUSG035570R167144	Slc22a7	chr17:46432184-46438477	2.34643	66.6019	-4.82703	0.00005	down
ENSMUSG035570R124766	Lipo1	chr19:33555159-33769142	11.3165	45.1941	-1.9977	0.00005	down
ENSMUSG035570R166071	Cyp4a12a	chr4:115299045-115332815	31.5218	83.3769	-1.4033	0.00005	down
ENSMUSG035570R139519	Cyp7b1	chr3:18071949-18243338	14.2507	52.1945	-1.87286	0.00005	down
ENSMUSG035570R144249	Defb29	chr2:152538713-152540098	185.62	550.861	-1.56934	0.00005	down
ENSMUSG035570R134875	Nudt19	chr7:35547184-35556304	161.669	714.768	-2.14443	0.00005	down
ENSMUSG035570R130004	Nat8	chr6:85830387-85832082	149.96	402.774	-1.42539	0.00005	down
ENSMUSG035570R117929	B4galt5	chr2:167298443-167349183	13.2999	35.6871	-1.42399	0.00005	down
ENSMUSG035570R129482	Aacs	chr5:125475813-125517410	26.2474	75.816	-1.53033	0.00005	down
ENSMUSG035570R126839	Upp2	chr2:58567386-58792971	1.30337	11.337	-3.12072	0.00005	down
ENSMUSG035570R135031	C8a	chr4:104815678-104876398	4.78809	13.7461	-1.5215	0.00005	down
ENSMUSG035570R138704	Aspdh	chr7:44465390-44467757	26.7967	77.3053	-1.52852	0.00005	down
ENSMUSG035570R128655	Mfsd2a	chr4:122946849-122961188	6.73343	25.4395	-1.91765	0.00005	down
ENSMUSG035570R129311	Hsd17b11	chr5:103989761-104021919	102.92	406.895	-1.98314	0.00005	down
ENSMUSG035570R152562	Slc22a30	chr19:8335370-8405111	27.5059	99.3218	-1.85237	0.00005	down
ENSMUSG035570R100673	Haao	chr17:83831355-83846790	89.7654	214.166	-1.2545	0.00005	down
ENSMUSG035570R103477	Inmt	chr6:55170625-55175043	402.509	1552.55	-1.94754	0.00005	down
ENSMUSG035570R157425	Ugt2b37	chr5:87240492-87254804	63.0667	486.022	-2.94607	0.00005	down
ENSMUSG035570R122244	Amacr	chr15:10981755-10996624	76.2004	258.821	-1.76409	0.00005	down

**Table 3:** The detail information of the regulated miRNAs

sRNA	IRI_readcount	CON_readcount	Log2(foldchange)	P value
mmu-miR-132-3p	452.6447157	171.8318385	1.2823	0.035570R100012142
mmu-miR-17-5p	3797.290026	2839.295813	0.41137	0.000029253
mmu-miR-21a-5p	762691.8641	382628.4345	0.88782	0.000074131
mmu-miR-21a-3p	101.0577404	34.32596001	1.1891	0.00010339
mmu-miR-20a-5p	8679.716112	6517.37373	0.40251	0.00049414
mmu-miR-93-5p	6005.296397	5013.213568	0.25767	0.00060655
mmu-miR-185-5p	7013.596268	8487.749035	-0.27186	0.00091314
mmu-miR-874-3p	1020.061146	1308.48056	-0.35206	0.0011189

**Table 4:** The detail information of the regulated circRNAs

ID	IRI_readcount	CON_readcount	Log2(foldchange)	P value	Regulation
mmu_circ_0001548	14.21687218	0	5.9489	0.0005576	up
mmu_circ_0001956	8.422620727	0	5.2867	0.0031394	up
mmu_circ_0002196	5.545773914	0	4.7296	0.010686	up
mmu_circ_0004550	5.342042355	0	4.6872	0.011569	up
mmu_circ_0000103	32.73614474	0	4.854	0.013752	up
mmu_circ_0001489	5.552315004	0	4.5757	0.015308	up
mmu_circ_0006082	10.66964162	0	4.6802	0.01552	up
mmu_circ_0006225	7.359266568	0	4.5724	0.017181	up
mmu_circ_0001534	4.438566573	0	4.4294	0.018892	up
mmu_circ_0000745	4.846029692	0	4.3864	0.021489	up
mmu_circ_0004646	6.806318542	0	4.4008	0.02325	up
mmu_circ_0003372	6.436167516	0	4.3791	0.023835	up
mmu_circ_0002604	3.952340736	0	4.2881	0.024182	up
mmu_circ_0004758	3.846052751	0	4.2462	0.026045	up
mmu_circ_0005809	3.738822351	0	4.1917	0.028596	up
mmu_circ_0006467	176.2563137	48.53769451	1.7821	0.03061	up
mmu_circ_0006472	577.8870696	170.626607	1.6917	0.033934	up
mmu_circ_0009113	3.444188307	0	4.0837	0.034049	up
mmu_circ_0000481	3.876823753	0	4.0963	0.034579	up
mmu_circ_0005155	3.393843652	0	4.0681	0.034895	up
mmu_circ_0006487	0	15.39395838	-6.0144	0.0004595	down
mmu_circ_0004381	0	11.18513945	-5.6136	0.0013845	down
mmu_circ_0007639	0	5.728120787	-4.7428	0.010352	down
mmu_circ_0001815	0	6.351436042	-4.7055	0.011989	down
mmu_circ_0008801	0	22.99108264	-4.8628	0.01288	down
mmu_circ_0007839	8.180684277	54.68063599	-2.5423	0.015794	down
mmu_circ_0004158	0	5.553344879	-4.5436	0.016169	down
mmu_circ_0008707	0	4.406656193	-4.4121	0.019322	down
mmu_circ_0003583	0	4.323975941	-4.3844	0.020345	down
mmu_circ_0006770	0	4.322406707	-4.3737	0.020805	down
mmu_circ_0007841	5.552315004	34.8047102	-2.4456	0.02154	down
mmu_circ_0009173	0	4.247572625	-4.201	0.029118	down
mmu_circ_0004671	0	4.164892373	-4.1756	0.030356	down
mmu_circ_0008750	0	4.132415606	-4.1552	0.031376	down
mmu_circ_0000166	0	3.520084988	-4.1103	0.032366	down
mmu_circ_0001678	0	3.518515754	-4.1001	0.032987	down
mmu_circ_0003806	0	3.877005827	-4.0658	0.036137	down
mmu_circ_0009847	0	3.337940057	-4.007	0.038363	down
mmu_circ_0004698	0	3.331663122	-4.005	0.038481	down
mmu_circ_0006648	0	2.903046668	-3.8512	0.048287	down

### Validation of ncRNAs and mRNAs expression via quantitative polymerase chain reaction (qPCR)

**Figure 3:**
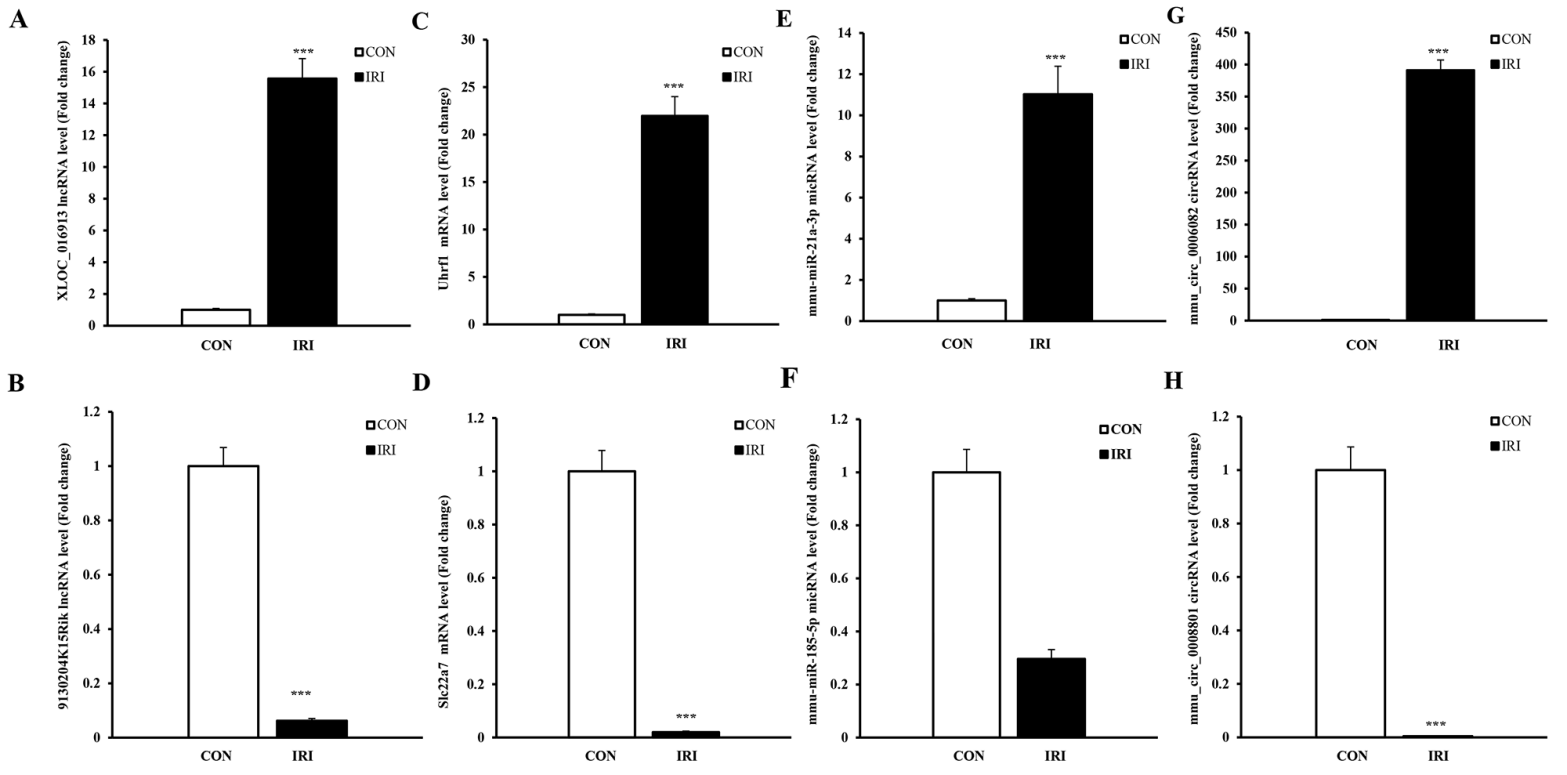
qPCR validations of eight regulated ncRNAs in the kidneys of mice subjected to IR The expression levels of lncRNAs **(A** and **B)** showed significantly different levels at 24 hours in kidneys from mice in the IRI group compared with mice in the CON group. The expression levels of mRNAs **(C** and **D)** showed significantly different levels at 24 hours in the kidneys of mice from the IRI group compared with those from the CON group. The expression levels of miRNAs **(E** and **F)** showed significantly different levels at 24 hours in the kidneys of mice from the IRI group compared with CON group. The expressions of lncRNAs **(G** and **H)** showed significantly different levels at 24 hours in the kidneys of mice from the IRI group compared with CON group. One-way ANOVA followed by Tukey’s multiple comparison test. ^***^P < 0.001.

To validate the reliability of the sequencing results and provide the basis for further study, eight RNAs among the DE ncRNA and mRNA transcripts were randomly selected to validate the accuracy of the sequencing data using qPCR, including 2 lncRNAs, 2 circRNAs, 2 miRNAs and 2 mRNAs. Figure 3 shows that all of the selected ncRNA and mRNA transcripts were detected and exhibited significantly different expression in the kidneys of mice subjected to IR. These results were consistent with the RNA sequencing data.

### Functional prediction of DE ncRNAs in IR induced-AKI

To ascertain the functions and connections of the differentially expressed genes in IR-induced AKI, we performed Gene Ontology (GO) and Kyoto Encyclopedia of Genes and Genomes (KEGG) analyses with an absolute value of correlation greater than 0.95.

**Figure 4:**
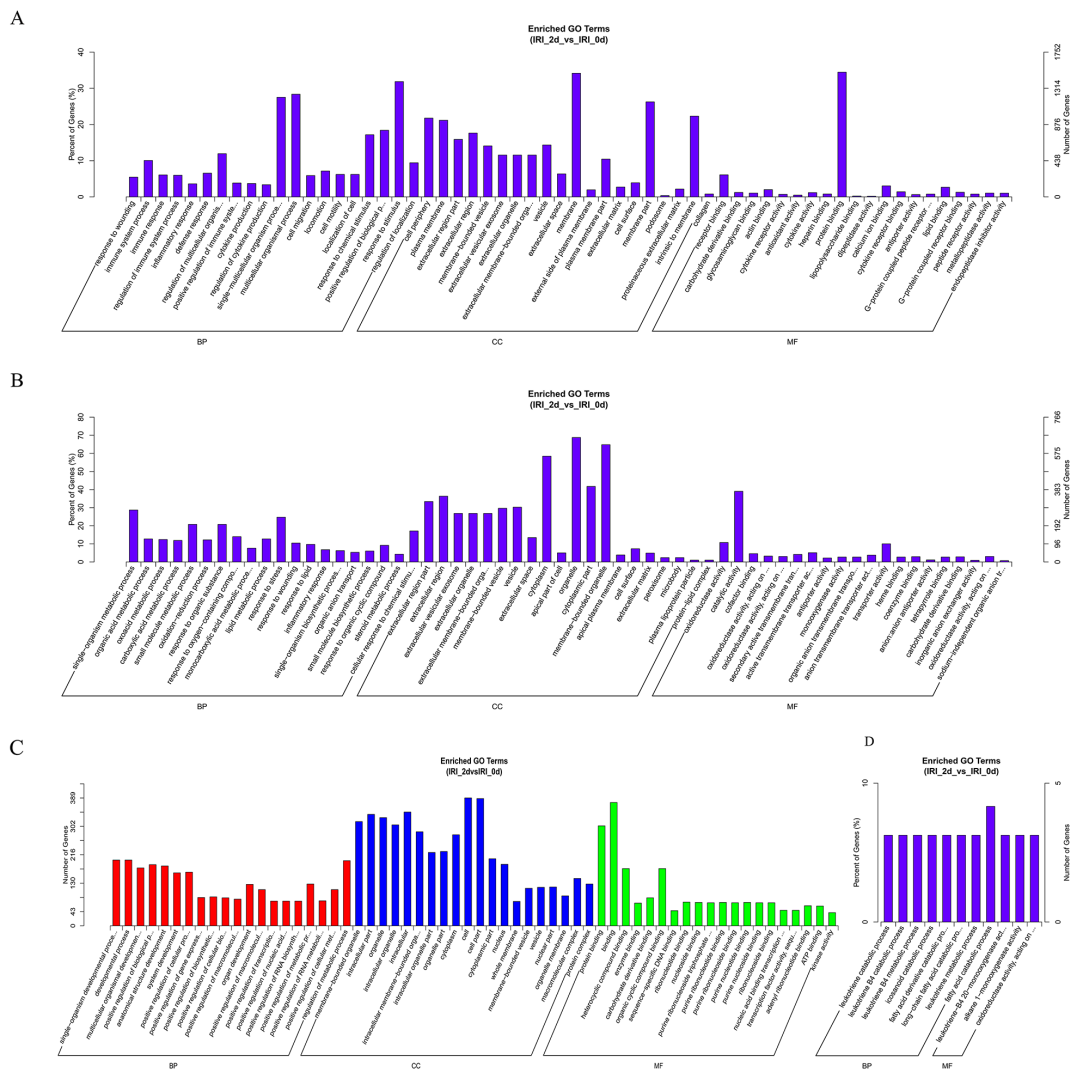
GO analysis of lncRNAs, mRNAs, miRNAs and circRNAs in the kidneys of mice subjected to IR The significant MFs, BPs and CCs of genes associated with the DE lncRNAs in the kidneys of mice subjected to IR are presented. The GO term of DE lncRNAs co-expressed genes are shown in a histogram **(A)**. The GO term of predicted mRNAs in the kidneys of mice subjected to IR are shown in a histogram **(B)**. The GO term of DE miRNAs in the kidneys of mice subjected to IR are shown in **(C)**. The BP and MF GO terms of DE circRNAs in the kidneys of mice subjected to IR are shown in **(D)**.

GO (http://www.geneontology.org/) is the international standard classification system of gene function [19]. According to the distribution of the predicted target genes in the Gene Ontology analysis, the number of genes was statistically analyzed with significant enrichment of each GO term to clarify gene function in biological process (BP), cellular component (CC) and molecular function (MF), and the data are presented as a histogram. Based on the GO analysis of the co-located and co-expressed genes of the DE lncRNAs (Figure 4A), DE mRNA (Figure 4B), DE miRNA (Figure 4C) and DE circRNA (Figure 4D), the most enriched GO terms were listed in Table 5. These most striking category of gene function will indicate the direction for our further research of ncRNAs.

**Table 5:** The most enriched GO terms and KEGG pathway

	GO terms				KEGG Pathway
	BP	CC	MF		
**lncRNA**	Stimulus response Multicellular organismal processes Single-multicellular organism processes	Membrane Membrane part Intrinsic to the membrane	Protein binding Receptor binding Calcium ion binding	**lncRNA** **&** **mRNA**	Metabolic pathways Osteoclast differentiation The TNF signaling pathway
**mRNA**	Single-organism metabolic process Response to stress Response to organic substance	Organelles Membrane-bound organelles The cytoplasm	Oxidoreductase activity Catalytic activity Transportor activity		The p53 signaling pathway Pproteoglycans in cancer Pathways in cancer
**miRNA**	Single-organism developmental process Developmental process Regulation of metabolic process	Cell Cell part Intracellular	Binding Protein binding Organic cyclic compound binding	**miRNA** **&** **circRNA**	The MAPK signaling pathway Vascular smooth muscle contraction Retinol metabolism The PPAR signaling pathway
**circRNA**	Fatty acid catabolic process Leukotriene metabolic process Long-chain fatty acid catabolic process	\ \ \	Leukotriene-B4 20-monooxygenase activity Alkane 1-monooxygenase activity Oxidoreductase activity acting on NADH		Inflammatory mediator regulation of TRP channels Fatty acid elongation Arachidonic acid metabolism

**Figure 5:**
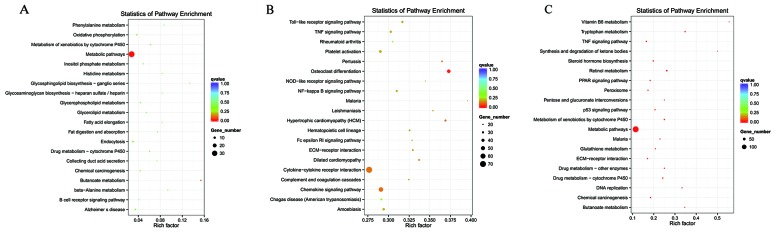
Enriched lncRNAs and mRNAs based on the KEGG pathway scatterplot of the RNA expression in the kidneys of mice subjected to IR lncRNA that co-localized with genes enriched in the KEGG pathway scatterplot indicating the statistics of pathway enrichment in the kidneys of mice subjected to IR **(A)**. lncRNAs that were co-expressed with genes enriched in the KEGG pathway scatterplot showing the statistics of pathway enrichment in the kidneys of mice subjected to IR **(B)**. Predicted mRNAs enriched in the KEGG pathway scatterplot showing the statistics of pathway enrichment in the kidneys of mice subjected to IR **(C)**.

**Figure 6:**
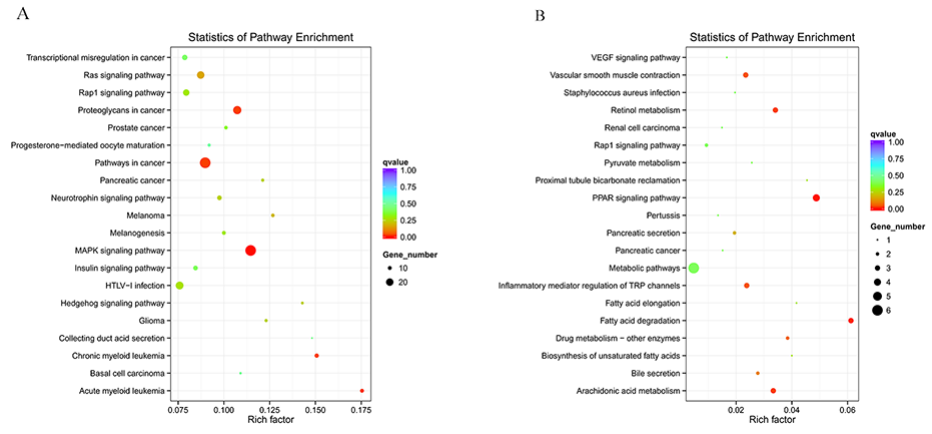
Enriched miRNAs and circRNAs based on the KEGG pathway scatterplot of the RNA expression in the kidneys of mice subjected to IR miRNAs enriched in the KEGG pathway scatterplot showing the statistics of pathway enrichment in the kidneys of mice subjected to IR **(A)**; circRNAs enriched in the KEGG pathway scatterplot showing the statistics of pathway enrichment in the kidneys of mice subjected to IR **(B)**.

KEGG is a collection of databases with information regarding genomes, biological pathways, diseases, drugs, and chemical substances; these databases can determine significantly enriched pathways among the candidate target genes compared with the entire genome background [20, 21]. The top 20 pathways enriched by the candidate target genes are displayed in an enriched scatter diagram, and the degree of KEGG enrichment is reported using the rich factor, q value and number of genes. When the rich factor is greater, q value is closer to zero, and the number of genes is bigger, the enrichment is more significant. Our results showed the most significantly involved pathways in IR-induced AKI based on the KEGG analysis of the intersection of co-localized and co-expressed genes of DE lncRNAs and predicted mRNAs (Figure 5A-5C) and DE miRNAs and DE circRNAs (Figure 6A, 6B). The most enriched GO terms and KEGG pathway were listed in Table 5. These main biochemical and signal transduction pathways will be the focus of future studies.

### Regulatory network of ncRNAs and mRNAs in IR-induced AKI

**Figure 7:**
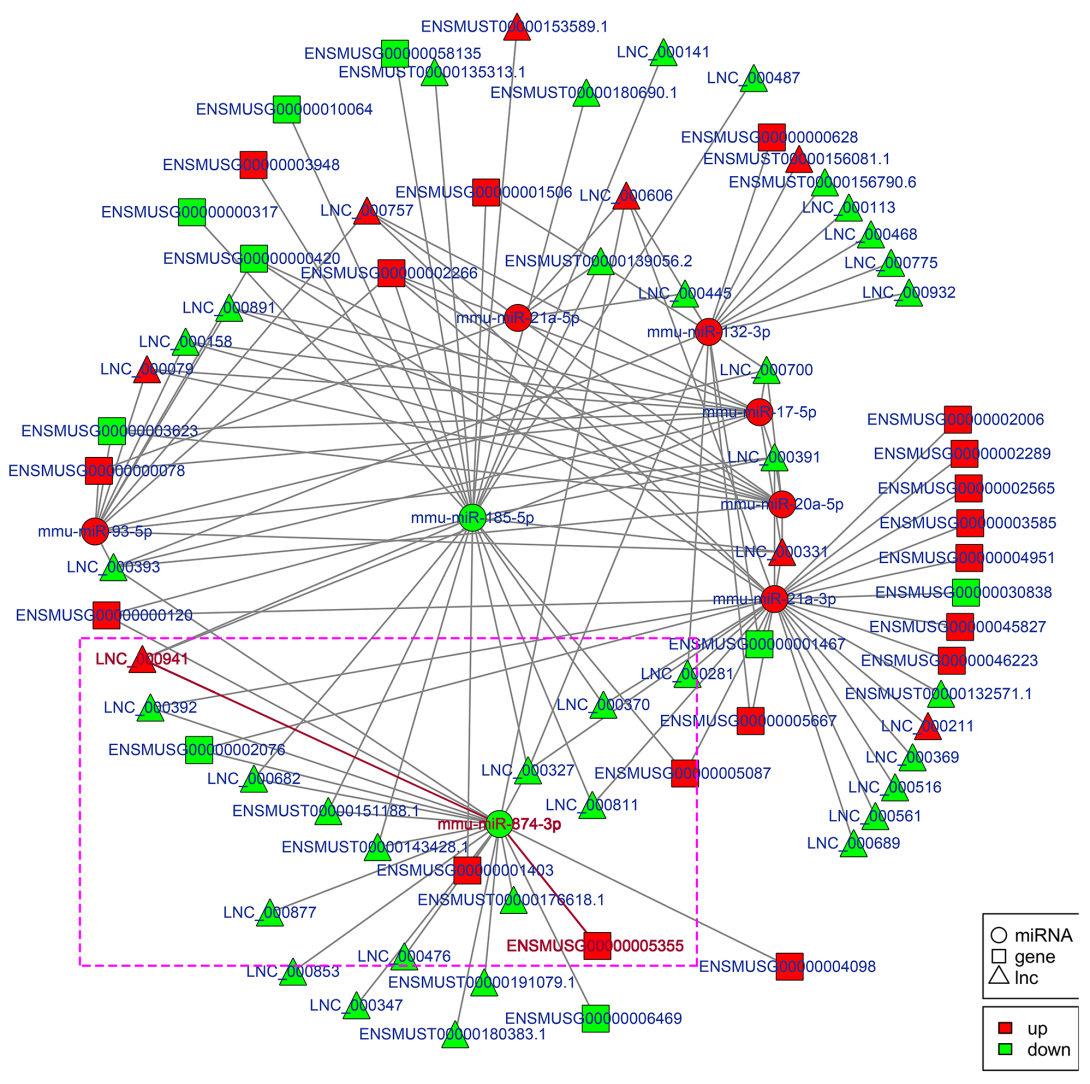
Regulatory network analysis of lncRNA-miRNA-mRNAs in the kidneys of mice subjected to IR Figure 7 shows the interactive network of lncRNA-miRNA-mRNAs in the kidneys of mice subjected to IR. mmu-miR-874-3p and LNC_000941 in purple box were verified with dual-luciferase reporter system in Figure 9.

**Figure 8:**
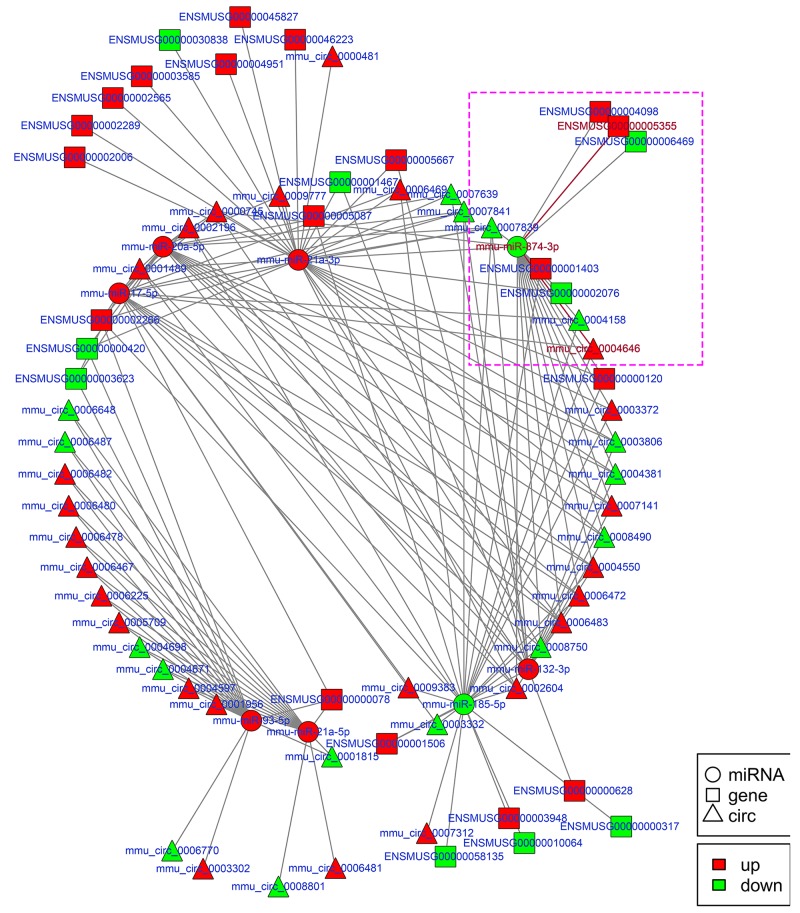
Regulatory network analysis of circRNA-miRNA-mRNAs in the kidneys of mice subjected to IR Figure 8 shows the interactive network of circRNA-miRNA-mRNAs in the kidneys of mice subjected to IR. mmu-miR-874-3p and mmu_circ_0004646 in purple box were verified with dual-luciferase reporter system in Figure 9.

To explore the molecular mechanism of ncRNAs involved in the pathogenesis of IR-induced AKI, we conducted an additional regulatory network analysis of ncRNAs and mRNAs. LncRNAs or circRNAs act as miRNA sponges to competitively interact with the binding sites of miRNAs, which play an extensive regulatory role [22, 23]. Therefore, a regulatory network of lncRNA-miRNA-mRNA pairs with lncRNA as a decoy, miRNA as the connector, and mRNA as the target is shown in Figure 7. A regulatory network of circRNA-miRNA-mRNA pairs with circRNA as a decoy, miRNA as the connector, and mRNA as the target is shown in Figure 8. The regulatory relationship of ncRNAs and mRNAs in the mechanism of IR-induced AKI was revealed through these regulatory networks. In fact, based on the above results, the regulatory role of ncRNAs in the pathogenesis of IR-induced AKI was so complicated that in-depth study should be implemented in the future.

### Verification of ncRNAs regulatory network

**Figure 9:**
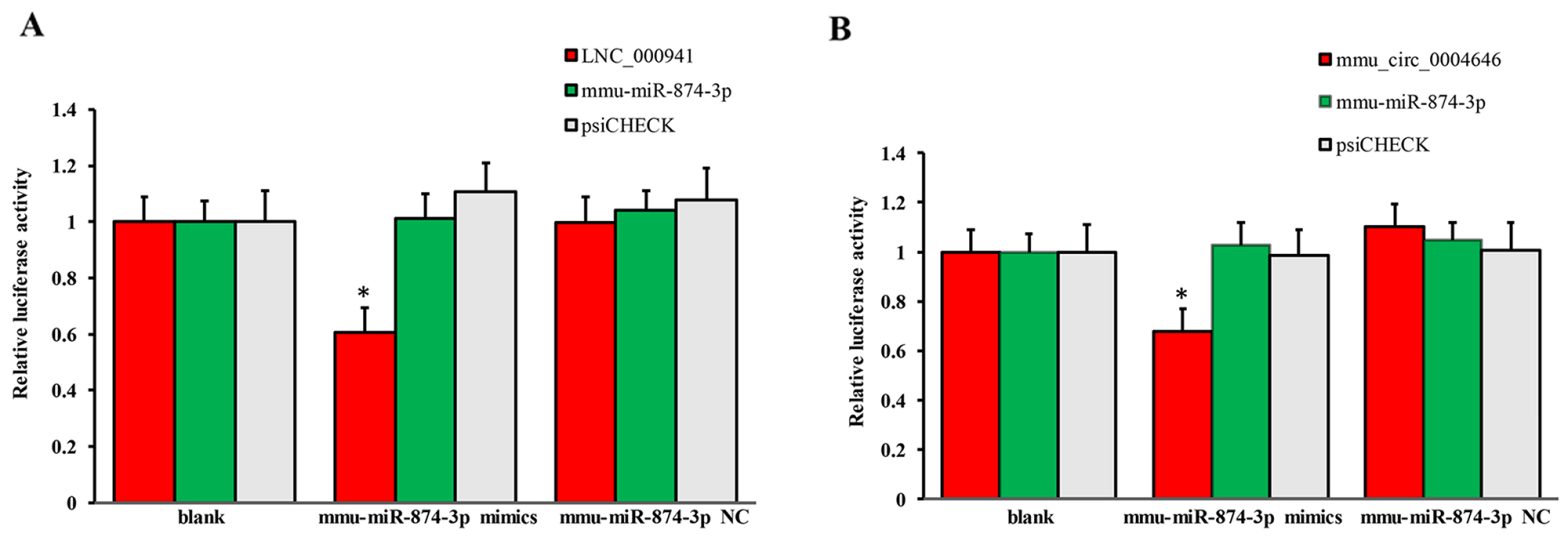
Confirmation of the pairs relationship Luciferase assays using reporter constructs lncRNA (LNC_000941) **(A)** or cirRNA (mmu_circ_0004646) **(B)** were performed in NSCs transfected with mmu-miR-874-3p (or a control). n = 5, ^*^p < 0.05 compared to the blank group.

Apoptosis of renal tubular epithelial cells play an important role in the procession of IR-induced AKI. We found that caspase14 (ENSMUSG035570R105355) was significantly upregulated in the kidneys of mice in the IRI group, which was proved to be an important anti-apoptotic protein [24]. Caspase14 was directly regulated by mmu-miR-874-3p, which was proved that over-expression promoted cellular apoptosis [25]. Therefore, considering that the most common mode of action with ncRNA pairs is sponging effect as ceRNA, we selected two pairs (mmu-miR-874-3p and LNC_000941, mmu-miR-874-3p and mmu_circ_0004646) to verify in renal tubular epithelial cells of mice with dual-luciferase reporter system. Luciferase assay revealed that mmu-miR-874-3p displayed a sponging effect for LNC_000941 (Figure 9A) and mmu_circ_0004646 (Figure 9B) and decreased luciferase activity. The results verified the accuracy of the network interaction of ncRNAs in Figure 7 and 8.

## DISCUSSION

IR are the main cause of AKI, which presents as impaired renal function, inflammation activity and apoptosis of the renal tubular epithelium [26–28]. In recent years, although there are many studies that have attempted to clarify the etiology and pathogenesis of IR-induced AKI, it is difficult to fully understand the underlying mechanism [29, 30]. Therefore, identifying the underlying mechanism is crucial to determine new therapeutic targets and personalize treatment methods. In the present study, this is the first overall report that showed ncRNAs and mRNAs in the kidney that underwent significant changes in response to IR-induced AKI. In addition, we also predicted the potential functions of DE ncRNAs by GO and KEGG analysis and constructed a regulatory network of ncRNAs and mRNAs in the kidneys of mice subjected to IR. With this knowledge, our findings on the transcription gene analysis provide us with an overall vision of ncRNAs in the pathogenesis of IR-induced AKI as well as useful clues for future and thorough research of the role of ncRNAs in AKI.

We prepared the mouse model of IR-induced AKI to analyze ncRNAs in our study. This model of IR-induced AKI guaranteed the sequencing results. According to the surgery protocol of the IRI model, the serum BUN and creatine levels in mice were tested at 24 hours after either IRI or sham procedures based on the theory that the deterioration of renal function occurs within 24 hours after IRI [31]. In accordance with the pathological results, the results of the renal function verified the successful preparation of this IRI model. In addition, apoptosis of renal tubular epithelial cells has been proven to be an important mechanism of IR-induced AKI by many scholars [29, 32]. We also observed TUNEL-positive cells in the kidneys of mice in the IRI and CON groups, which further verified the reliability of the IRI models.

NcRNAs play an important role in several fundamental biological and pathological processes and are associated with a variety of diseases [33, 34]. Earlier researchers commonly used microarrays to screen and predict DE ncRNAs in various pathophysiological processes [35]. To better clarify the overall changes and the role of ncRNAs in IR-induced AKI, we adopted the method of the second-generation sequencing. Although there are some limitations in our study, such as a relatively small sample size, we identified novel transcripts aside from annotated transcripts in databases. Moreover, the sensitive detection and reliable quantification of transcripts are the primary advantages of RNA sequencing compared with microarrays, and this method could identify ncRNAs that play important role but are expressed at low levels. In addition, eight DE transcripts identified in the present study were randomly selected to verify the accuracy of the RNA sequencing data by using qPCR. Ultimately, all the results were consistent with the RNA sequencing data, which confirmed again the reliability of our sequencing data and provided a credible base for further study.

Numerous findings have indicated that ncRNAs are involved in the cellular and molecular mechanisms of AKI by inducing multiple pathways [36, 37]. Some evidence has shown that the dominant role of miRNAs is to promote the pathological development of IR-induced AKI as determined by microarrays [40, 41], and recent studies also showed that lncRNAs were involved in the regulatory process of IR-induced AKI [11, 40]. However, there is little comprehensive knowledge regarding ncRNAs (i.e., miRNAs, lncRNAs and circRNAs) in IR-induced AKI. Therefore, we examined the DE ncRNAs and mRNAs in the kidneys of mice subjected to IR-induced AKI. Our results showed that a total of 90 lncRNAs, 8 miRNAs, 56 circRNAs and 993 mRNAs were significantly up-regulated or down-regulated in the kidney 24 hours after IR injury. These data are essential and provide the groundwork for a more thorough and comprehensive analysis of potential ncRNAs involved in IR-induced AKI.

To predict the potential functions of the DE ncRNAs identified in present study, GO and KEGG analyses were performed. GO terms and GO annotations are good predictors of gene function and can elucidate the genetic regulatory networks by forming hierarchical categories organized by molecular function, biological process, and cellular component [41]. The KEGG database is used to understand the high-level functions and utilities of the biological system [42].

The GO functional annotation analysis showed that these DE ncRNAs were enriched in several BPs (response to stimulus, multicellular organismal processes, single-multicellular organism processes, single-organism metabolic processes, stress responses, responses to organic substances, single-organism developmental processes, developmental processes, regulation of metabolic processes, fatty acid catabolic processes, leukotriene metabolic processes, long-chain fatty acid catabolic processes, fatty acid derivative catabolic processes, icosanoid catabolic processes, leukotriene B4 metabolic processes, leukotriene B4 catabolic processes, and leukotriene catabolic processes), CCs (membrane, partial membrane, intrinsic to membrane, organelles, membrane-bound organelles, the cytoplasm, whole cell, partial cell and intracellular areas), and MFs (oxidoreductase activity, catalytic activity, transportor activity, binding, protein binding, receptor binding, calcium ion binding, organic cyclic compound binding, leukotriene-B4 20-monooxygenase activity, alkane 1-monooxygenase activity and oxidoreductase activity and acting on NADH). Moreover, KEGG analysis showed that the main biochemical and signal transduction pathways were enriched in metabolic pathways, osteoclast differentiation, the TNF signaling pathway, the p53 signaling pathway, proteoglycans in cancer, pathways in cancer, the MAPK signaling pathway, vascular smooth muscle contraction, retinol metabolism, the PPAR signaling pathway, inflammatory mediator regulation of TRP channels, fatty acid elongation and arachidonic acid metabolism. The above mentioned gene functions and pathways of the predicted ncRNAs in the present data, were also shown in many previous studies about AKI. For example, large amount of previous studies provided evidence that oxidoreductase activity, membrane, etc. were closely associated with AKI [43-45]. Dagher PC proposed that activation of p53 are major inducers of apoptotic cell death after ischemic renal injury [46]. Huang W proved that lncRNA PVT1 promote AKI by regulating TNFα and JNK/NF-κB pathways [47]. There are plenty of evidence indicated that MAPK signaling pathway were involved in renal ischemia-reperfusion injury [48]. The conclusion in above previous studies can support our sequencing data.

The hypothesis of competing endogenous RNAs reveals a new interactive mechanism of RNA [49]. miRNAs can cause gene silencing by binding mRNAs [50], lncRNA, cirRNA, even mRNA could serve as ceRNA, can competitively bind to miRNAs to regulate gene expression via miRNA response elements (MREs). The interactive networks of ncRNAs that regulate mRNAs reveal the important role of ncRNA function, which has biological significance [51, 52]. Our data respectively showed the interactive network of lncRNA-miRNA-mRNA and circRNA-miRNA-mRNA, which play a regulatory role as observed with mmu-miR-132-3p, mmu-miR-17-5p, mmu-miR-21a-5p, mmu-miR-21a-3p, mmu-miR-20a-5p, mmu-miR-93-5p, mmu-miR-185-5p and mmu-miR-874-3p. It is worth mentioning that miR-21, miR-223-5p, miR-125b and so on proved to be involved in IR-induced AKI, did not appear in our data [53-55]. The reason is that all ncRNA sequencing analysized were based on the lncRNA library, not built separate library of miRNA. Therefore, comparing the sequencing method of single building library, DE miRNAs in our data had certain omissions. In future study, combined with previous studies and related database, we can compensate the missing miRNAs in our data. Although little is known about the role of circRNAs in IR-induced AKI, we presented a reliable direction of study for circRNAs.

Although evidence has accumulated showing that ncRNAs have significant role in the pathogenesis of AKI in the past few years, the molecular mechanisms underlying the interaction of ncRNAs in AKI remain largely unclear. It has been well demonstrated that miRNAs can function as negative regulators of gene expression in the initiation and/or progression stages of AKI. Therefore, the lncRNA-miRNA-mRNA and circRNA-miRNA-mRNA network of IR-induced AKI were constructed based on the theory of ceRNA, which lncRNAs or circRNAs act as natural miRNA sponges to suppress miRNA function using shared MREs for mutual regulation. These pioneering discoveries might enrich understanding on the mechanisms underlying the role of ncRNAs in the pathogenesis of AKI. For example, miR-132-3p and miR-17-5p were proved to be associated with inflammatory [56, 57], miR-185-5p and miR-874-3p are involved in apoptosis in response to damage [25, 58]. While it is consensus of experts that inflammatory and apoptosis are imporatant factors in AKI. Therefore, further developed and more targeted study can be done to explore how these ncRNAs mediated AKI by mechanism of ceRNA based on the lncRNA-miRNA-mRNA and circRNA-miRNA-mRNA network in combination with the raw data in SRA.

In conclusion, the present study revealed for the first time that ncRNAs are significantly altered in IR-induced AKI based on second-generation sequencing data. In addition, the data indicated that aberrantly expressed ncRNAs participate in the interaction and regulation of the expression of related genes and are involved in related specific biological processes and pathways that may contribute to the pathogenesis of AKI. While our findings provide newfound and full-scaled information regarding the critical role of ncRNAs in IR-induced AKI, further research is required to fully elucidate the detailed molecular mechanisms underlying the DE ncRNAs in our dataset that have a predicted function.

## MATERIALS AND METHODS

### Animals

Adult male BALB/c mice (10 to 12 weeks old age, body weight 25-30 g, the Laboratory Animal Center of The First People’s Hospital of Foshan, Foshan, China) were randomly assigned to either the IRI group or the CON group (6 animals per group). All animal procedures were in accordance with national and international animal care and ethical guidelines and have been approved by the institutional animal welfare committee. The environment was maintained at a constant temperature (22±0.581°C) and relative humidity (60-70%) with a 12-hour light/dark cycle (lights on at 7 AM). All animals were provided standard laboratory chow and tap water ad libitum. Implementation of the IRI model is described below. Mice were anesthetized by intraperitoneal injection of ketamine (80 mg/kg) and xylazine (10 mg/kg). Kidneys were exposed through a flank incision and were subjected to ischemia by clamping the renal pedicles using non-traumatic microaneurysm clamps. After 30 min, the clamps were removed, and blood flow was reestablished. Body temperature was maintained at 36.5-37.5°C throughout the entire procedure. Mice in the CON group underwent an identical surgical procedure but without pedicle clamping. All the animals were sacrificed at 24 hours after reperfusion, and the kidneys were harvested.

### Measurement of renal function

Serum creatinine was measured using a creatinine assay kit (BioAssay Systems, Hayward, CA) according to the manufacturer’s instructions. Blood urea nitrogen was determined fluorometrically as previously described [59].

### Renal morphology

Kidney tissue was fixed with 10% buffered formalin, embedded in paraffin, and sliced into sections 4-μm-thick. After deparaffinization and rehydration, the sections were stained with either hematoxylin and eosin or periodic Acid Schiff (PAS). Tissue damage was examined in a blinded manner and scored according to the percentage of damaged tubules: 0, no damage; 1, less than 25% damage; 2, 25%-50% damage; 3, 50%-75% damage; and 4, more than 75% damage as previously reported [60].

### Detection of apoptotic cells

Apoptotic cell death was determined by using terminal deoxynucleotidyl transferase-mediated dUTP nick-end labeling (TUNEL) staining with the DeadEnd Colorimetric Apoptosis Detection System (Millipore, Billerica, MA) according to manufacturer’s instructions. The number of TUNEL-positive cells per high-power field were counted and analyzed in a blinded fashion.

### Quantitative real-time RT-PCR

Total RNA was extracted from kidney tissues using TRIzol reagent (Invitrogen). Aliquots (1 μg) of total RNA were reverse transcribed using SuperScript II reverse transcriptase. Real-time PCR was performed using the IQ SYBR green SuperMix reagent (Bio-Rad, Herculus, CA) with a Bio-Rad real-time PCR machine according to the manufacturer’s instructions. The comparative Ct method (ΔΔCt) was used to quantify gene expression, and the relative quantification was calculated as 2^−ΔΔCt^. The expression levels of the target genes were normalized to the GAPDH levels in each corresponding sample. The primer sequences are listed in Table 6.

**Table 6:** Primers designed for qRT-PCR validation of candidate ncRNAs and mRNAs

Gene	Primer	Product Length(bp)
LNC000424	F: CCTGACTTCTCACCAGAATC R: GGCTGACATCTGTGATCTCT	81
ENSMUST00000150312.1	F: CATCTGTCACGGTGTTTGG R: TGGGTTTGAGTCTCCAGGAT	140
Uhrf1	F: TCAGTGAGTCCGGTGTGCAT R: TGTACGCTTGTTGCCAGAGA	170
Slc22a7	F: ACTGCCCAAACTTGCTTATG R: GCTAATTCAGTCCCGGATCT	150
mmu_circ_0006082	F: CTGAATGGGGCCAGGTTCTC R: CATGTGCTGTCCTTGCATAG	196
mmu_circ_0008801	F: GGGATCAGGCAGAGGATGAC R: ATCATGGTCCGCCTATGCTT	199
mmu-miR-185-5p	F: ACACTCCAGCTGGGTGGAGAGAAAGGCAGTTC R: CTCAACTGGTGTCGTGGA	72
mmu-miR-21a-3p	F: ACACTCCAGCTGGGCAACAGCAGTCGATGGGC R: CTCAACTGGTGTCGTGGA	72
U6	F: CTCGCTTCGGCAGCACA R: AACGCTTCACGAATTTGCGT	94
β-actin	F: GCTTCTAGGCGGACTGTTAC R: CCATGCCAATGTTGTCTCTT	100

### Tissue collection and RNA isolation

We prepared twelve mice for either IR or a sham operation, and all animals were deeply anesthetized with isoflurane at 24 hours after undergoing IRI or the sham operation. Total RNA was extracted from the kidney tissue using TRIzol reagent (Invitrogen, Carlsbad). RNA degradation and contamination was monitored using 1% agarose gels. RNA purity was measured using a NanoPhotometer® spectrophotometer (IMPLEN, CA, USA). The RNA concentration was measured using a Qubit® RNA Assay kit and a Qubit® 2.0 Fluorometer (Life Technologies, CA, USA). RNA integrity was assessed using a RNA Nano 6000 Assay kit with a Bioanalyzer 2100 system (Agilent Technologies, CA, USA).

### Library preparation for ncRNA sequencing

A total of 3 μg of RNA per sample was used as input material for the RNA sample preparations of lncRNA sequencing. First, ribosomal RNA was removed using a Epicenter Ribo-zero™ rRNA Removal Kit (Epicenter, USA), and rRNA-free residue was washed by ethanol precipitation. Subsequently, sequencing libraries were generated using an rRNA-depleted RNA by NEBNext® Ultra™ Directional RNA Library Prep kit for Illumina® (NEB, USA) following the manufacturer’s recommendations. Sequencing libraries of small RNA were generated using an NEBNext® Multiplex Small RNA Library Prep Set for Illumina® (NEB, USA) following manufacturer’s recommendations, and index codes were added to the attribute sequences in each sample [61].

### Clustering and sequencing of ncRNA

The clustering of the index-coded samples was performed on a cBot Cluster Generation System using a TruSeq PE Cluster Kit v3-cBot-HS (Illumina) according to the manufacturer’s instructions. After cluster generation, the libraries were sequenced on an Illumina HiSeq 2500 platform, and 125 bp paired-end and 50 bp single-end reads were generated. The transcription with splicing of each sample were combined and screened as lncRNAs with Cuffmerge Software, and the conditions were as follows: the number of exon≥2, length > 200 bp, FPKM ≥0.5 (Cuffquant) and to eliminate overlapping and coding potential transcription with annotation of database at exon region (Cuffcompare Software). CircRNAs were identified base on the data of lncRNAs with find_circ [62]. Clean reads were screened the lengh of 21–22 nt as miRNA, and located to reference sequence with bowtie. Combined with miREvo Software and mirdeep2 Software to analysis the funtions of new miRNAs. Adopt DESeq2 with negative binomial distribution to analyse differentially expression of ncRNAs. All sequencing program were performed by Novogene Company (China, Beijing).

### GO and KEGG analysis

GO and KEGG analysis were applied to investigate the roles of all the DE ncRNAs. In brief, GO analysis was applied to elucidate the genetic regulatory networks of interest by forming hierarchical categories according to the BP, CC and MF of the differentially expressed genes (http://www.geneontology.org). Pathway analysis was performed using KEGG (http://www.genome.jp/kegg/) to explore the significant pathways of the differentially expressed genes.

### Analysis of the ncRNA regulatory networks

Interactive networks were built and visualized using Cytoscape software based on the screened lncRNA-miRNA gene pairs and the circRNA-miRNA gene pairs. Different shapes represent the different types of RNA, whereas the different colors represent the regulated relationship. The size of the node was directly proportion to extent of association. In other words, these significant nodes are in a core position in the regulated network and were more associated with IR-induced AKI.

### Luciferase assay

A dual-luciferase reporter system E1960 (Promega, Madison, WI, USA) was used to perform luciferase activity assay. In brief, renal tubular epithelial cell of mouse were cultured on 12-well tissue culture plates at a density of 2 × 10^5^ cells per well. Cells were co-transfected with the luciferase reporter constructs contain lncRNA (LNC_000941) or cirRNA (mmu_circ_0004646), miRNA(mmu-miR-874-3p) mimics and Renilla luciferase construct for 5h(Lipofectamine® MessengerMAX™ Transfection Reagent, Thermo Fisher Scientific). After 3d culture at 37°C, the transfected cells were lysed by 150 μl of passive lysis buffer. In total, 30 μl of lysates were mixed with 50 μl of LAR II, and then firefly luciferase activity was measured by a luminometer. For the internal control, 50 μl of Stop & Glo reagent was added to the sample.

### Statistical analysis

The data are presented as the means±SEM. The results from the behavioral study were statistically analyzed using either one-way or two-way analysis of variance (ANOVA). The qPCR results were analyzed by one-way analysis of variance followed by Tukey’s multiple comparison test. Significance was set at p<0.05.

## References

[1] Bomsztyk K, Denisenko O (2013). Epigenetic alterations in acute kidney injury. Semin Nephrol.

[2] Rewa O, Bagshaw SM (2014). Acute kidney injury-epidemiology, outcomes and economics. Nat Rev Nephrol.

[3] Hoste EA, Kellum JA, Katz NM, Rosner MH, Haase M, Ronco C (2010). Epidemiology of acute kidney injury. Contrib Nephrol.

[4] Agarwal A, Dong Z, Harris R, Murray P, Parikh SM, Rosner MH, Kellum JA, Ronco C, Acute Dialysis Quality Initiative XIII Working Group (2016). Cellular and molecular mechanisms of AKI. J Am Soc Nephrol.

[5] Anathhanam S, Lewington AJ (2013). Acute kidney injury. J R Coll Physicians Edinb.

[6] Fliser D, Laville M, Covic A, Fouque D, Vanholder R, Juillard L, Van Biesen W, The ad-hoc working group of ERBP (2012). A European Renal Best Practice (ERBP) position statement on the kidney disease improving global outcomes (KDIGO) clinical practice guidelines on acute kidney injury: part 1: definitions, conservative management and contrast-induced nephropathy. Nephrol Dial Transplant.

[7] Mattick JS, Makunin IV (2006). Non-coding RNA. Hum Mol Genet.

[8] Thum T, Condorelli G (2015). Long noncoding RNAs and microRNAs in cardiovascular pathophysiology. Circ Res.

[9] Godwin JG, Ge X, Stephan K, Jurisch A, Tullius SG, Iacomini J (2010). Identification of a microrna signature of renal ischemia reperfusion injury. Proc Natl Acad Sci U S A.

[10] Wang KC, Chang HY (2011). Molecular mechanisms of long noncoding RNAs. Mol Cell.

[11] Thum T (2014). Noncoding RNAs and myocardial fibrosis. Nat Rev Cardiol.

[12] Yu TM, Palanisamy K, Sun KT, Day YJ, Shu KH, Wang IK, Shyu WC, Chen P, Chen YL, Li CY (2016). RANTES mediates kidney ischemia reperfusion injury through a possible role of HIF-1α and LncRNA PRINS.. Sci Rep.

[13] Li Z, Deng X, Kang Z, Wang Y, Xia T, Ding N, Yin Y (2016). Elevation of miR-21, through targeting MKK3, may be involved in ischemia pretreatment protection from ischemia-reperfusion induced kidney injury. J Nephrol.

[14] Bellinger MA, Bean JS, Rader MA, Heinz-Taheny KM, Nunes JS, Haas JV, Michael LF, Rekhter MD (2014). Concordant changes of plasma and kidney microRNA in the early stages of acute kidney injury: time course in a mouse model of bilateral renal ischemia-reperfusion. PLoS One.

[15] Munshi R, Hsu C, Himmelfarb J (2011). Advances in understanding ischemic acute kidney injury. BMC Med.

[16] Mehta RL, Pascual MT, Soroko S, Savage BR, Himmelfarb J, Ikizler TA, Paganini EP, Chertow GM, Program to Improve Care in Acute Renal Disease (2004). Spectrum of acute renal failure in the intensive care unit: the PICARD experience. Kidney Int.

[17] Jin X, Chen J, Hu Z, Chan L, Wang Y (2013). Genetic deficiency of adiponectin protects against acute kidney injury. Kidney Int.

[18] Liang H, Zhang Z, He L, Wang Y (2016). CXCL16 regulates cisplatin-induced acute kidney injury. Oncotarget.

[19] Young MD, Wakefield MJ, Smyth GK, Oshlack A (2010). Gene ontology analysis for RNA-seq: accounting for selection bias. Genome Biol.

[20] Hashimoto K, Goto S, Kawano S, Aoki-Kinoshita KF, Ueda N, Hamajima M, Kawasaki T, Kanehisa M (2006). KEGG as a glycome informatics resource. Glycobiology.

[21] Kanehisa M, Goto S, Sato Y, Kawashima M, Furumichi M, Tanabe M (2014). Data, information, knowledge and principle: back to metabolism in KEGG. Nucleic Acids Res.

[22] Ren K, Li Y, Lu H, Li Z, Li Z, Wu K, Li Z, Han X (2016). Long noncoding RNA HOTAIR controls cell cycle by functioning as a competing endogenous RNA in esophageal squamous cell carcinoma. Transl Oncol.

[23] Kulcheski FR, Christoff AP, Margis R (2016). Circular RNAs are miRNA sponges and can be used as a new class of biomarker. J Biotechnol.

[24] Chamcheu JC, Pal HC, Siddiqui IA, Adhami VM, Ayehunie S, Boylan BT, Noubissi FK, Khan N, Syed DN, Elmets CA, Wood GS, Afaq F, Mukhtar H (2015). Prodifferentiation, anti-inflammatory and antiproliferative effects of delphinidin, a dietary anthocyanidin, in a full-thickness three-dimensional reconstituted human skin model of psoriasis. Skin Pharmacol Physiol.

[25] Leong KW, Cheng CW, Wong CM, Ng IO, Kwong YL, Tse E (2017). miR-874-3p is down-regulated in hepatocellular carcinoma and negatively regulates PIN1 expression. Oncotarget.

[26] Malek M, Nematbakhsh M (2015). Renal ischemia/reperfusion injury; from pathophysiology to treatment. J Renal Inj Prev.

[27] Jang HR, Ko GJ, Wasowska BA, Rabb H (2009). The interaction between ischemia–reperfusion and immune responses in the kidney. J Mol Med (Berl).

[28] Yang Y, Song M, Liu Y, Liu H, Sun L, Peng Y, Liu F, Venkatachalam MA, Dong Z (2016). Renoprotective approaches and strategies in acute kidney injury. Pharmacol Ther.

[29] Sharfuddin AA, Molitoris BA (2011). Pathophysiology of ischemic acute kidney injury. Nat Rev Nephrol.

[30] Malek M, Nematbakhsh M (2015). Renal ischemia/reperfusion injury; from pathophysiology to treatment. J Renal Inj Prev.

[31] Hao J, Wei Q, Mei S, Li L, Su Y, Mei C, Dong Z (2017). Induction of microRNA-17-5p by p53 protects against renal ischemia-reperfusion injury by targeting death receptor 6. Kidney Int.

[32] Sanz AB, Santamaria B, Ruiz-Ortega M, Egido J, Ortiz A (2008). Mechanisms of renal apoptosis in health and disease. J Am Soc Nephrol.

[33] Caiment F, Gaj S, Claessen S, Kleinjans J (2015). High-throughput data integration of RNA–miRNA–circRNA reveals novel insights into mechanisms of benzo[a]pyrene-induced carcinogenicity. Nucleic Acids Res.

[34] Wang W, Gao Z, Wang H, Li T, He W, Lv W, Zhang J (2016). Transcriptome analysis reveals distinct gene expression profiles in eosinophilic and noneosinophilic chronic rhinosinusitis with nasal polyps. Sci Rep.

[35] Lee DY, Moon J, Lee ST, Jung KH, Park DK, Yoo JS, Sunwoo JS, Byun JI, Shin JW, Jeon D, Jung KY, Kim M, Lee SK (2015). Distinct expression of long non-coding RNAs in an Alzheimer’s disease model. J Alzheimers Dis.

[36] Khalid U, Bowen T, Fraser DJ, Jenkins RH (2014). Acute kidney injury: a paradigm for miRNA regulation of the cell cycle. Biochem Soc Trans.

[37] Zhou P, Chen Z, Zou Y, Wan X (2016). Roles of non-coding RNAs in acute kidney injury. Kidney Blood Press Res.

[38] Cui R, Xu J, Chen X, Zhu W (2016). Global miRNA expression is temporally correlated with acute kidney injury in mice. Peer J.

[39] Wang JF, Zha YF, Li HW, Wang F, Bian Q, Lai XL, Yu G (2014). Screening plasma miRNAs as biomarkers for renal ischemia-reperfusion injury in rats. Med Sci Monit.

[40] Lorenzen JM, Schauerte C, Kielstein JT, Hübner A, Martino F, Fiedler J, Gupta SK, Faulhaber-Walter R, Kumarswamy R, Hafer C, Haller H, Fliser D, Thum T (2015). Circulating long noncoding RNATapSaki is a predictor of mortality in critically ill patients with acute kidney injury. Clin Chem.

[41] Camon E, Magrane M, Barrell D, Lee V, Dimmer E, Maslen J, Binns D, Harte N, Lopez R, Apweiler R (2004). The Gene Ontology Annotation (GOA) Database: sharing knowledge in Uniprot with Gene Ontology. Nucleic Acids Res.

[42] Du J, Li M, Yuan Z, Guo M, Song J, Xie X, Chen Y (2016). A decision analysis model for KEGG pathway analysis. BMC Bioinformatics.

[43] Haga Y, Ohtsubo T, Murakami N, Noguchi H, Kansui Y, Goto K, Matsumura K, Kitazono T (2017). Disruption of xanthine oxidoreductase gene attenuates renal ischemia reperfusion injury in mice. Life Sci.

[44] Geng X, Hong Q, Wang W, Zheng W, Li O, Cai G, Chen X, Wu D (2017). Biological membrane-packed mesenchymal stem cells treat acute kidney disease by ameliorating mitochondrial-related apoptosis. Sci Rep.

[45] Fan Y, Xiao W, Lee K, Salem F, Wen J, He L, Zhang J, Fei Y, Cheng D, Bao H, Liu Y, Lin F, Jiang G (2017). Inhibition of reticulon-1A-mediated endoplasmic reticulum stress in early AKI attenuates renal fibrosis development. J Am Soc Nephrol.

[46] Dagher PC (2004). Apoptosis in ischemic renal injury: roles of GTP depletion and p53. Kidney Int.

[47] Huang W, Lan X, Li X, Wang D, Sun Y, Wang Q, Gao H, Yu K (2017). Long non-coding RNA PVT1 promote LPS-induced septic acute kidney injury by regulating TNFα and JNK/NF-κB pathways in HK-2 cells. Int Immunopharmacol.

[48] Chen J, Wang W, Zhang Q, Li F, Lei T, Luo D, Zhou H, Yang B (2013). Low molecular weight fucoidan against renal ischemia-reperfusion injury via inhibition of the MAPK signaling pathway. PLoS One.

[49] Salmena L, Poliseno L, Tay Y, Kats L, Pandolfi PP (2011). A ceRNA hypothesis: the Rosetta stone of a hidden RNA language?. Cell.

[50] Thomas M, Lieberman J, Lal A (2010). Desperately seeking microRNA targets. Nat Struct Mol Biol.

[51] Khalil AM, Guttman M, Huarte M, Garber M, Raj A, Rivea Morales D, Thomas K, Presser A, Bernstein BE, van Oudenaarden A, Regev A, Lander ES, Rinn JL (2009). Many human large intergenic noncoding RNAs associate with chromatin-modifying complexes and affect gene expression. Proc Natl Acad Sci U S A.

[52] Guttman M, Amit I, Garber M, French C, Lin MF, Feldser D, Huarte M, Zuk O, Carey BW, Cassady JP, Cabili MN, Jaenisch R, Mikkelsen TS (2009). Chromatin signature reveals over a thousand highly conserved large non-coding RNAs in mammals. Nature.

[53] Li Z, Deng X, Kang Z, Wang Y, Xia T, Ding N, Yin Y (2016). Elevation of miR-21, through targeting MKK3, may be involved in ischemia pretreatment protection from ischemia-reperfusion induced kidney injury. J Nephrol.

[54] Colbert JF, Ford JA, Haeger SM, Yang Y, Dailey KL, Allison KC, Neudecker V, Evans CM, Richardson VL, Brodsky KS, Faubel S, Eltzschig HK, Schmidt EP (2017). A model-specific role of microRNA-223 as a mediator of kidney injury during experimental sepsis. Am J Physiol Renal Physiol.

[55] Wang X, Ha T, Zou J, Ren D, Liu L, Zhang X, Kalbfleisch J, Gao X, Williams D, Li C (2014). MicroRNA-125b protects against myocardial ischaemia/reperfusion injury via targeting p53-mediated apoptotic signalling and TRAF6. Cardiovasc Res.

[56] Rider CF, Yamamoto M, Günther OP, Hirota JA, Singh A, Tebbutt SJ, Carlsten C (2016). Controlled diesel exhaust and allergen coexposure modulates microRNA and gene expression in humans: Effects on inflammatory lung markers. J Allergy Clin Immunol.

[57] Hasan HF, Abdel-Rafei MK, Galal SM (2016). Diosmin attenuates radiation-induced hepatic fibrosis by boosting PPAR-γ expression and hampering miR-17-5p-activated canonical Wnt-β-catenin signaling. Biochem Cell Biol.

[58] Schauerte C, Hübner A, Rong S, Wang S, Shushakova N, Mengel M, Dettling A, Bang C, Scherf K, Koelling M, Melk A, Haller H, Thum T (2017). MicroRNA-185-5p restores glucocorticoid sensitivity by suppressing the mammalian target of rapamycin complex (mTORC) signaling pathway to enhance glucocorticoid receptor autoregulation. Kidney Int.

[59] Ramesh G, Zhang B, Uematsu S, Akira S, Reeves WB Endotoxin and cisplatin synergistically induce renal dysfunction and cytokine production in mice. Am J Physiol Renal Physiol.

[60] Chen G, Lin SC, Chen J, He L, Dong F, Xu J, Han S, Du J, Entman ML, Wang Y (2011). CXCL16 recruits bone marrow-derived fibroblast precursors in renal fibrosis. J Am Soc Nephrol.

[61] Zhou J, Xiong Q, Chen H, Yang C, Fan Y (2017). Identification of the spinal expression profile of non-coding RNAs involved in neuropathic pain following spared nerve injury by sequence analysis. Front Mol Neurosci.

[62] Memczak S, Jens M, Elefsinioti A, Torti F, Krueger J, Rybak A, Maier L, Mackowiak SD, Gregersen LH, Munschauer M, Loewer A, Ziebold U, Landthaler M (2013). Circular RNAs are a large class of animal RNAs with regulatory potency. Nature.

